# Enteroendocrine Cells Are Specifically Marked by Cell Surface Expression of Claudin-4 in Mouse Small Intestine

**DOI:** 10.1371/journal.pone.0090638

**Published:** 2014-03-06

**Authors:** Takahiro Nagatake, Harumi Fujita, Nagahiro Minato, Yoko Hamazaki

**Affiliations:** Department of Immunology and Cell Biology, Graduate School of Medicine, Kyoto University, Kyoto, Japan; Emory University School of Medicine, United States of America

## Abstract

Enteroendocrine cells are solitary epithelial cells scattered throughout the gastrointestinal tract and produce various types of hormones, constituting one of the largest endocrine systems in the body. The study of these rare epithelial cells has been hampered by the difficulty in isolating them because of the lack of specific cell surface markers. Here, we report that enteroendocrine cells selectively express a tight junction membrane protein, claudin**-**4 (Cld4), and are efficiently isolated with the use of an antibody specific for the Cld4 extracellular domain and flow cytometry. Sorted Cld4^+^ epithelial cells in the small intestine exclusively expressed a chromogranin A gene (*Chga*) and other enteroendocrine cell–related genes (*Ffar1*, *Ffar4*, *Gpr119*), and the population was divided into two subpopulations based on the activity of binding to *Ulex europaeus* agglutinin**-**1 (UEA**-**1). A Cld4^+^UEA**-**1**^−^** cell population almost exclusively expressed glucose**-**dependent insulinotropic polypeptide gene (*Gip*), thus representing K cells, whereas a Cld4^+^UEA**-**1**^+^** cell population expressed other gut hormone genes, including glucagon**-**like peptide 1 (*Gcg*), pancreatic polypeptide**–**like peptide with N**-**terminal tyrosine amide (*Pyy*), cholecystokinin (*Cck*), secretin (*Sct*), and tryptophan hydroxylase 1 (*Tph1*). In addition, we found that orally administered luminal antigens were taken up by the solitary Cld4^+^ cells in the small intestinal villi, raising the possibility that enteroendocrine cells might also play a role in initiation of mucosal immunity. Our results provide a useful tool for the cellular and functional characterization of enteroendocrine cells.

## Introduction

The intestinal epithelial cell layer consists of functionally heterogeneous cell populations, including absorptive epithelial cells, goblet cells, paneth cells, M cells, cup cells, tuft cells, and enteroendocrine cells, all of which are derived from Lgr5^+^ crypt base columnar stem cells [1]–[3]. Among these, enteroendocrine cells include more than 10 different cell types producing distinct hormones or hormone-like substances, such as serotonin, secretin, glucose**-**dependent insulinotropic polypeptide (GIP), glucagon**-**like peptide 1 (GLP**-**1), pancreatic polypeptide**–**like peptide with N**-**terminal tyrosine amide, and cholecystokinin [4]. Those producing GIP and GLP**-**1 are called K cells and L cells [5], [6], respectively, and are responsible for “incretin effects” regulating postprandial insulin secretion [7]–[11]. Although enteroendocrine cells comprise only a small population of the intestinal epithelial cells, they function as essential regulators of digestion, appetite, gut motility, and metabolism, making the gut one of the largest endocrine organ in the body [12]. Several key transcriptional factors for the differentiation of enteroendocrine cells have been identified [13], [14]. However, how the cell lineages diverge and inter**-**relate remain poorly understood, in part because of the lack of specific cell markers for purifying live enteroendocrine cells. It has been reported that *Ulex europaeus* agglutinin**-**1 (UEA**-**1) binds some enteroendocrine cells, but it also reacts with other epithelial cell components, such as M cells in the follicle-associated epithelium (FAE) and exocrine goblet cells [15]. More recently, transgenic mice carrying fluorescent reporters under the control of *Gcg* and *Gip* promoters have enabled the identification and isolation of L cells and K cells, respectively [16], [17]. Nonetheless, general cell surface markers for the enteroendocrine cell population have not been identified.

Claudins (Clds), integral membrane proteins with four transmembrane domains, are crucial components of tight junctions (TJs) that function as a primary barrier to solutes and water as well as charge-selective channels between the apical and basal sides of epithelial cellular sheets [18], [19]. The Cld gene family comprises at least 24 members in mice and in humans [19]–[21]. Typically, multiple Clds are expressed in most types of epithelial cells, and the combination and ratio of different types of Clds in TJ strands may determine the permeability of each epithelial cellular sheet [20], [22]. Recent studies have revealed that Clds may also be involved in nonbarrier functions such as the regulation of cell proliferation and cell signaling [23]–[29]. A Cld family member, Cld4, may be one of these unique types of Clds. We previously reported that Cld4 is expressed in various TJ-deficient cells, such as thymic epithelial cells and developing T cells [28], [30]. In the intestinal mucosa, Cld4 is expressed in a portion of the tips of villi and FAE of the Peyer’s patches [31]–[33], providing a molecular target for drug delivery of the efficient mucosal vaccine [34]–[36]. In the current study, we demonstrate that Cld4 is selectively and abundantly expressed on the cell surface of enteroendocrine cells and serves as an effective molecular marker for their identification and isolation.

## Results

### Selective Expression of Cld4 in Intestinal Solitary Epithelial Cells Displaying Chromogranin A

**Figure 1:**
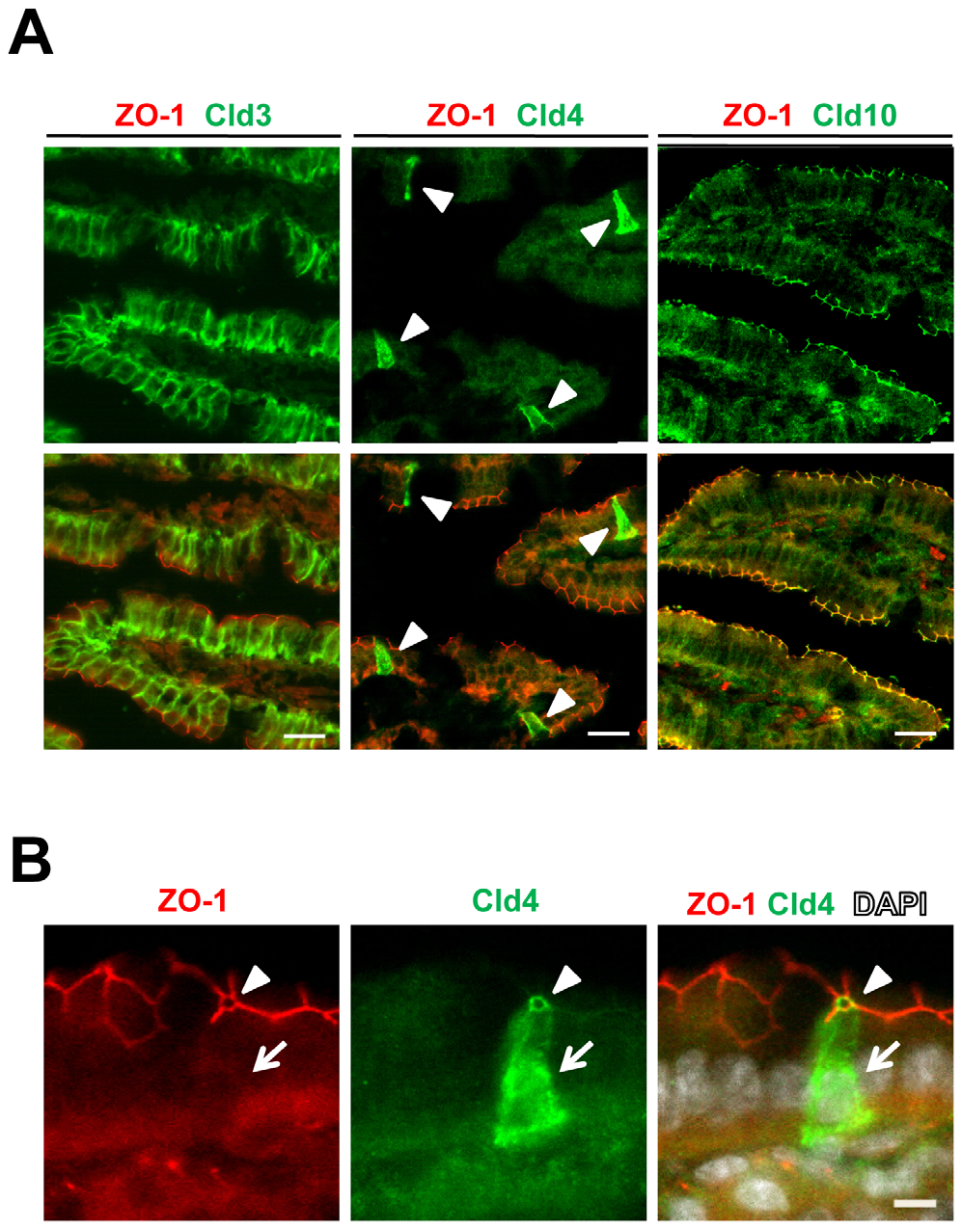
Expression of Cld4 in the solitary epithelial cells of the small intestinal villi. (*A*) Cryostat sections of the villus epithelia of 8-week-old to 10**-**week**-**old WT mice were fixed with 95% ethanol for 30 min at 4°C, followed by 100% acetone for 1 minute at room temperature before blocking and two-color immunostained with rabbit anti-Cld3, anti-Cld4, or anti-Cld10 polyclonal antibodies (green), along with rat anti-ZO-1 monoclonal antibody (red). Arrowheads indicate the spindle**-**shape solitary epithelial cells expressing Cld4. Bars, 20 µm. (*B*) Enlarged magnification of ZO-1 and Cld4 staining of villus epithelium. Cld4 expression is detected at TJs with the adjacent epithelial cells colocalizing with ZO-1 (arrowheads) in addition to the entire surface of the solitary epithelial cells (arrows). The results are representative of at least three independent experiments. Bar, 5 µm.

**Figure 2:**
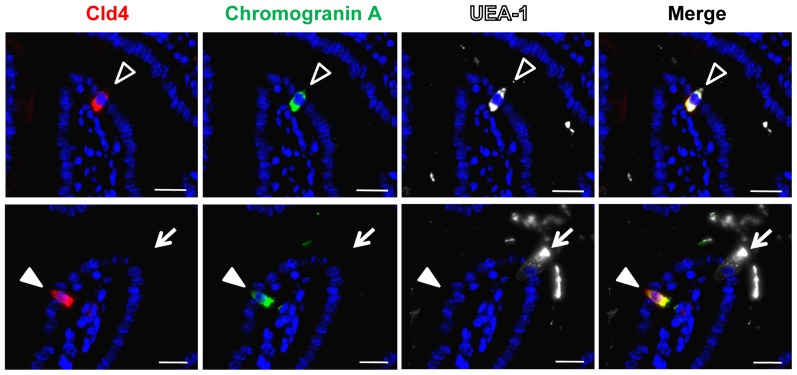
Solitary Cld4^+^ cells scattered in the intestinal villi express chromogranin A with or without UEA-1 binding. Villus epithelium was three-color immunostained with rat anti-Cld4 (HKH-189) (red), rabbit anti-chromogranin A (green) antibodies, and UEA-1 (white). Spindle**-**shape Cld4^+^ cells coexpressed chromogranin A. Some of the Cld4^+^ cells were costained with UEA**-**1 (open arrowheads), whereas the others were not (solid arrowheads). UEA-1^+^Cld4^−^ cells did not express chromogranin A (arrows). The results are representative of at least five independent experiments. Bars, 20 µm.

It was reported that several types of Clds are expressed in epithelial cells of mouse small intestine, including Cld3, Cld4, and Cld10 [24]. The expression of Cld10 was sharply concentrated at cell–cell contact sites of an entire epithelial cell sheet at the most apical region of the plasma membrane, colocalizing with ZO-1 (Figure 1A), suggesting that Cld10 expression is confined to TJs. Although Cld3 was also localized at cell–cell borders of the epithelial cellular sheet, the expression was much broader, covering entire basolateral regions (Figure 1A). In contrast, Cld4 expression was detected in rare and solitary cells scattered within the epithelial cellular sheet of the intestinal villi (Figure 1A). In these cells, Cld4 was localized diffusely and strongly throughout the entire cell surface in addition to the concentrated localization at ZO-1^+^ TJs formed with neighboring epithelial cells (Figure 1B). The characteristic immunostaining pattern was confirmed with the use of an independent rat monoclonal antibody that recognizes the extracellular domain of Cld4 (HKH-189) [28] (Figure S1). The signal with either antibody was completely absent in the intestine of *Cldn4* ^−/−^ mice [29] (Figure S1), confirming the specificity of the signal. The solitary Cld4^+^ cells showed a spindle**-**shape morphology (Figure 1A and B) reminiscent of enteroendocrine cells; therefore, we performed coimmunostaining of Cld4 with chromogranin A (CgA), a general marker of enteroendocrine cells [4] and UEA**-**1, which binds to goblet cells and some enteroendocrine cells [15]. We found that almost all of the solitary Cld4^+^ cells coexpressed CgA, irrespective of UEA**-**1 binding (Figure 2). On the other hand, UEA**-**1^+^CgA^−^ cells, most likely representing goblet cells, did not exhibit Cld4 expression (Figure 2). The results suggest that Cld4 is selectively expressed in enteroendocrine cells of small intestinal villi.

### The Expression of Enteroendocrine Cell–Associated Genes Is Confined to Isolated Cld4^+^ Intestinal Epithelial Cells

**Figure 3:**
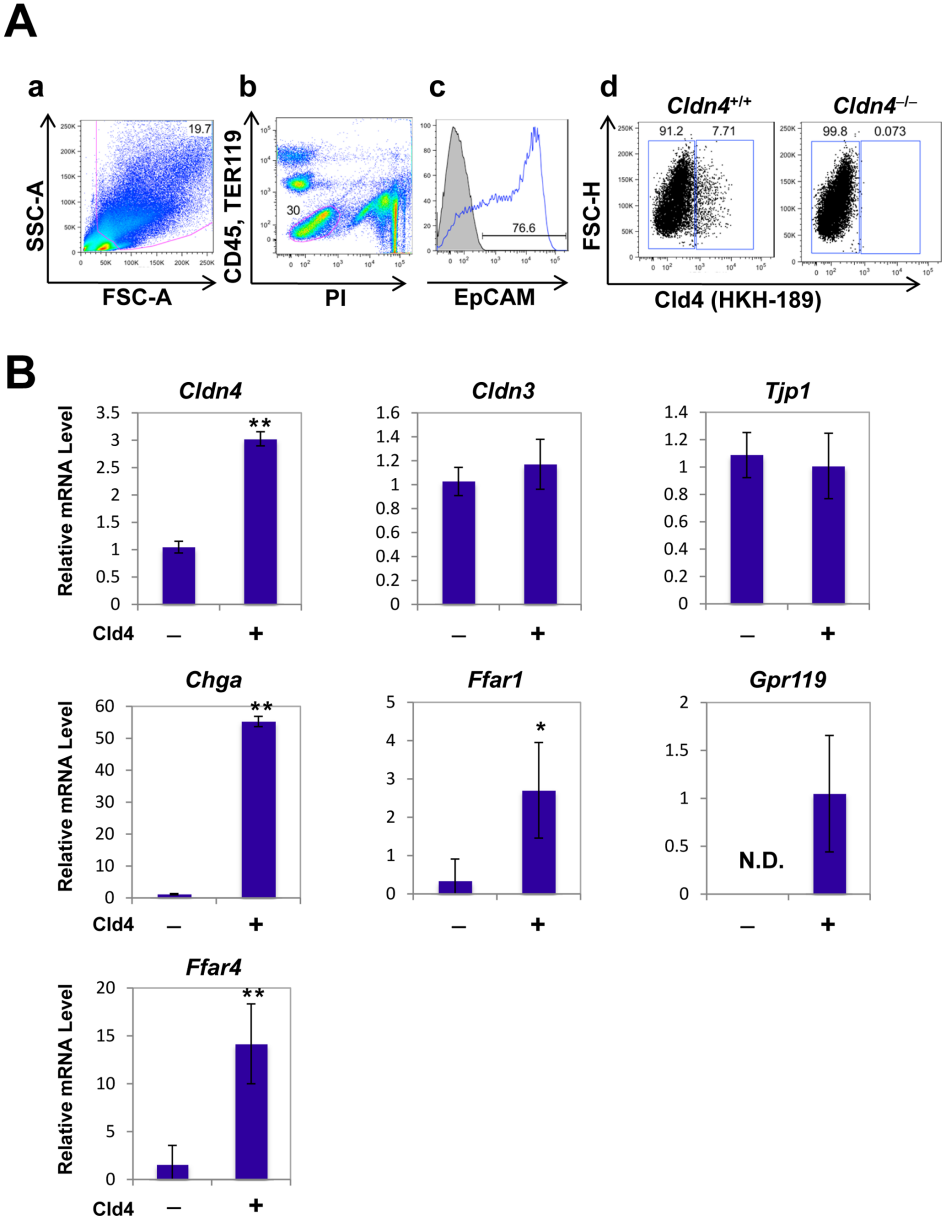
Cld4^+^ epithelial cells isolated by a cell-sorter with anti-Cld4 antibody exclusively express the genes related to enteroendocrine cells. (*A*) Multicolor FACS analysis of small intestine single-cell suspension from WT and *Cldn4* ^−/−^ mice. The profile of EpCAM expression in a PI^−^CD45^−^Ter119^−^ viable nonhematopoietic cell gate is shown (a−c). In the EpCAM^+^ cell gate, FACS profiles of biotinylated anti-Cld4 antibody (HKH-189) are indicated for WT and *Cldn4* ^−/−^ mice (d). Percentages of positive staining and negative staining are shown. (*B*) Cld4^+^ and Cld4^−^ fractions of EpCAM^+^ cells were sorted separately with FACSAria and examined for the transcripts of claudin-4 (*Cldn4*), claudin-3 (*Cldn3*), ZO-1 (*Tjp1*), chromogranin A (*Chga*), Gpr40 (*Ffar1*), Gpr119 (*Gpr119*), and Gpr120 (*Ffar4*) with quantitative RT-PCR. These results are representative of at least three independent experiments. *P < 0.05 and **P < 0.01, Student *t* test.

To confirm the features of Cld4^+^ solitary epithelial cells, we isolated them with a cell sorter using the rat anti-Cld4 mAb (HKH-189). Fluorescence-activated cell sorting (FACS) analysis revealed that approximately 8% of the total viable epithelial cell fraction (PI^−^CD45^−^Ter119^−^EpCAM^+^) of the intestinal single-cell preparation from wild-type (WT) mice showed positive staining with anti-Cld4 mAb (HKH-189), whereas virtually no positive signal was detected in that from *Cldn4* ^−/−^ mice (Figure 3A). We then separately sorted Cld4^+^ and Cld4^−^ fractions with a cell sorter. A Cld4^+^ fraction showed three-times more *Cldn4* transcripts than a Cld4^−^ fraction, whereas both cell fractions contained comparable levels of ZO**-**1(*Tjp1*) and Cld3 (*Cldn3*) transcripts (Figure 3B). In agreement with the immunostaining analysis, the transcripts of the chromogranin A gene (*Chga*) were confined exclusively to the Cld4^+^ cell fraction (Figure 3B). It has been reported that G**-**protein-coupled receptors, such as GPR40, GPR119, and GPR120, are specifically expressed in enteroendocrine cells and are involved in sensing free fatty acids, leading to the release of incretin hormones [37]–[39]. The transcripts of all these G-protein-coupled receptor genes (*Ffar1*, *Gpr119*, *Ffar4*) were also expressed preferentially in the Cld4^+^ cell fraction (Figure 3B). The results indicate that Cld4^+^ cells isolated with a cell sorter are highly enriched with all the enteroendocrine cells in the small intestine.

### Physical Separation of GIP-Producing K Cells From Enteroendocrine Cells Producing Other Peptide Hormones, Including GLP-1–Producing L Cells

**Figure 4:**
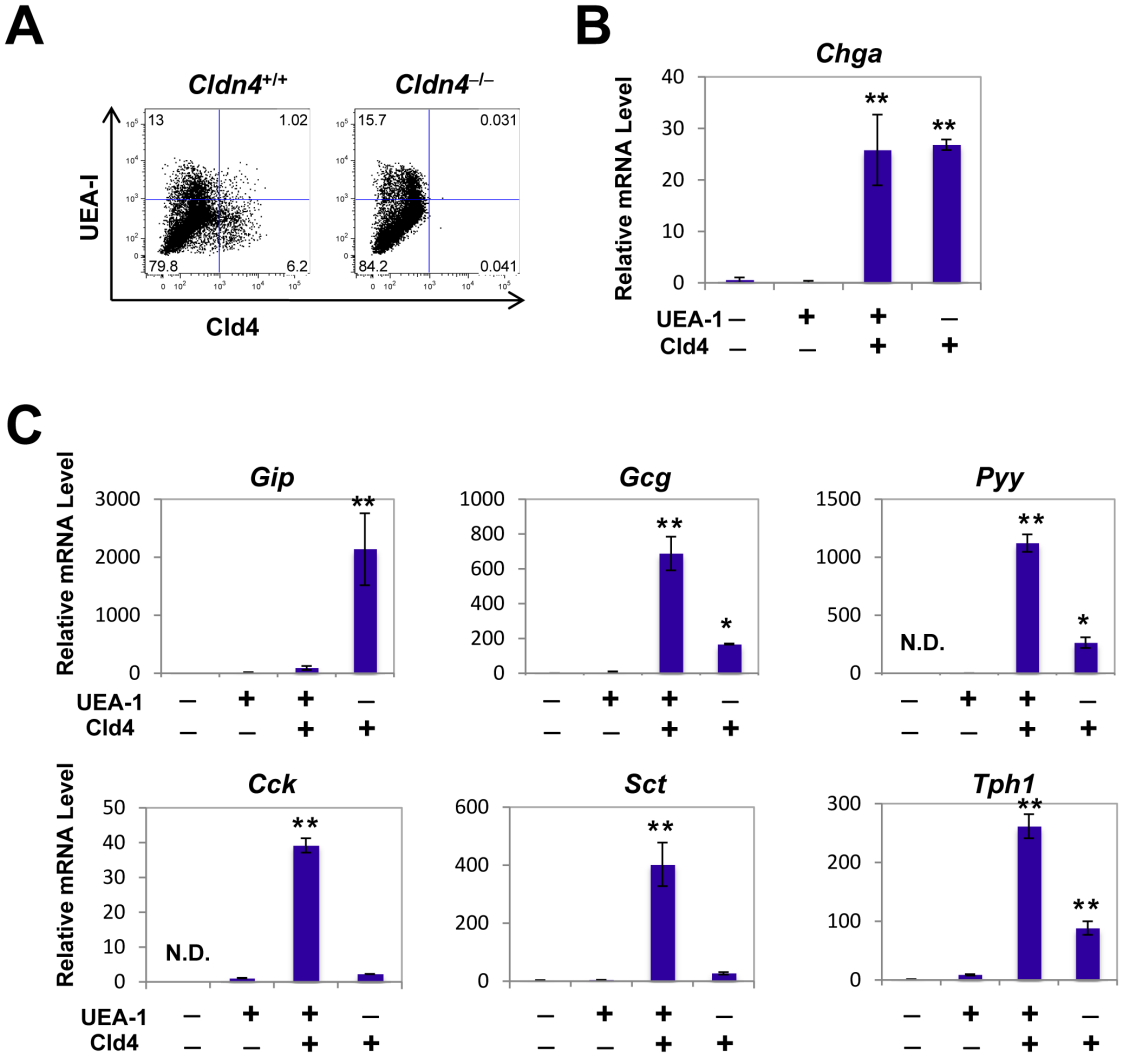
Cld4 expression and UEA*-*1 reactivity define the distinct enteroendocrine cell populations. (*A*) FACS profiles of biotinylated anti-Cld4 antibody (HKH-189) staining and UEA-1 binding in CD45^−^Ter119^−^PI^−^EpCAM^+^ intestinal epithelial cells from *Cldn4* ^+/+^ and *Cldn4* ^−/−^ mice are indicated. (*B*, *C*) Each epithelial cell population of four quadrant fractions in (*A*) of *Cldn4* ^+/+^ mice was sorted separately with FACSAria and examined for the expressions of the indicated genes with quantitative RT**-**PCR. The mean relative transcripts and standard errors are shown. The results are representative of at least three independent experiments. *P < 0.01 and **P < 0.001, ANOVA.

**Figure 5:**
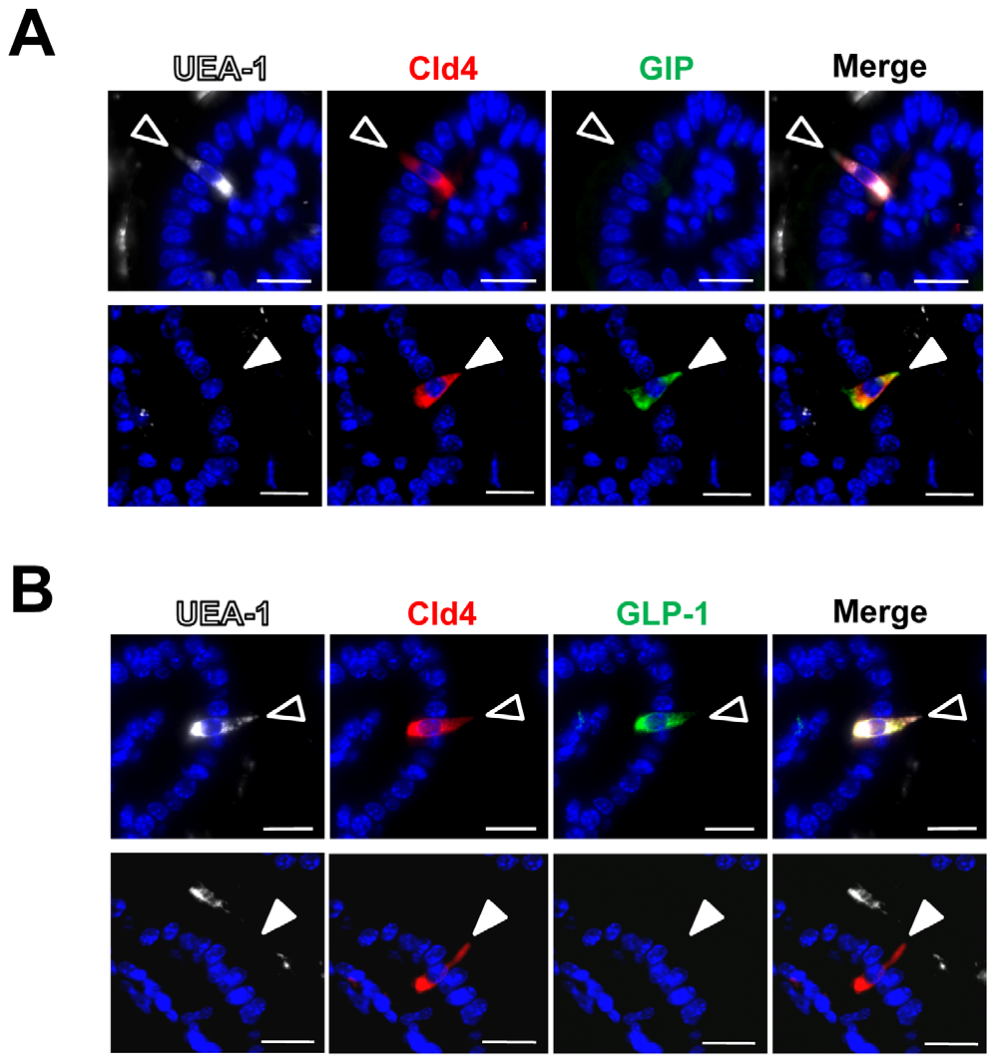
Cld4^+^UEA-1^−^ and Cld4^+^UEA-1^+^ cells express GIP and GLP-1 respectively. Small intestinal epithelia of 8-week-old to 10**-**week**-**old WT mice were multicolor-immunostained with rat anti-Cld4 antibody (HKH-189) (red), UEA-1 (white), and rabbit anti-GIP (*A*) or anti-GLP-1 (*B*) antibody (green). Open and closed arrowheads indicate UEA**-**1^+^Cld4^+^ and UEA**-**1^−^Cld4^+^ cells, respectively. The results are representative of at least five independent experiments. Bars, 20 µm.

By using multicolor FACS analysis with anti-Cld4 antibody (HKH-189) and UEA-1, we were able to identify four distinct populations in the intestinal EpCAM^+^ epithelial cells: Cld4^−^UEA-1^−^ cells (80%); Cld4^−^UEA-1^+^ cells (13%); Cld4^+^UEA-1^−^ cells (6%); and Cld4^+^UEA-1^+^ cells (1%) (Figure 4A). We then separately isolated the four populations with a cell sorter and examined the gene expression. Cld4^−^ populations hardly expressed *Chga* transcripts, irrespective of UEA-1 expression (Figure 4B). It was likely that Cld4^−^UEA-1^−^ and Cld4^−^UEA-1^+^ cells represented absorptive epithelial cells and goblet/M cells [15], respectively. On the other hand, both UEA-1^−^ and UEA-1^+^ populations within the Cld4^+^ fraction expressed comparable amounts of *Chga* transcripts, indicating that both fractions contained enteroendocrine cells (Figure 4B). Among the genes encoding representative intestinal peptide hormones, *Gip* was expressed exclusively in the Cld4^+^UEA-1^−^ cell fraction, whereas other genes, including *Gcg*, *Pyy*, *Cck*, *Sct*, and *Tph1*, were preferentially expressed in the Cld4^+^UEA**-**1^+^ cell fraction (Figure 4C). In particular, the expression of *Cck* and *Sct* was essentially exclusive to the Cld4^+^UEA**-**1^+^ cell fraction. As expected, Cld4^−^ cell fractions, either UEA**-**1^+^ or UEA**-**1^−^, exhibited no detectable expression of any of these enterohormone genes (Figure 4C). We confirmed the results at the protein level with immunostaining analysis. Expression of GIP was associated with UEA**-**1^−^, but not with UEA**-**1^+^, Cld4^+^ cells (Figure 5A), whereas GLP-1 expression was detected in a portion of Cld4^+^ UEA**-**1^+^ cells (Figure 5B). By immunohistochemical quantification in the small intestine, 85% (34/40) of Cld4^+^UEA-1^−^ cells was positive for GIP staining, whereas 68% (39/57) of Cld4^+^UEA-1^+^ cells expressed GLP-1. On the other hand, the expression of GIP and GLP-1 was also detected in a minor fraction of Cld4^+^UEA-1^+^ (3/30; 10%) and Cld4^+^UEA-1^−^ (7/54; 13%) cells, respectively, suggesting an overlap of endocrine hormone expression as reported previously [17], [40], [41]. Nonetheless, these results indicate that viable GIP**-**producing K cells are physically separable from other enteroendocrine cells including GLP-1–producing L cells by means of a flow cytometric cell sorter.

### Transepithelial Passage of Luminal Antigen by Cld4^+^ Enteroendocrine Cells

**Figure 6:**
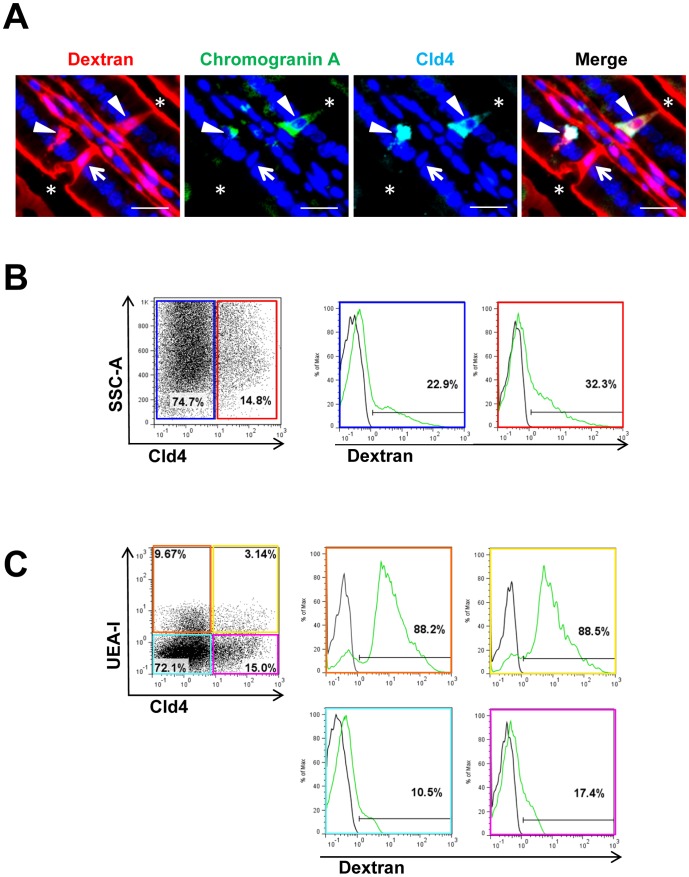
Transepithelial passage of luminal dextran (10-kDa) via Cld4^+^ enteroendocrine cells. Eight-week-old to 10**-**week**-**old WT mice were administered oral rhodamine or fluorescein**-**conjugated 10-kDa dextran and euthanized 30 minutes later. (*A*) The small intestines were immunostained with rabbit anti-chromogranin A (green) and rat anti-Cld4 (HKH-189) (blue) antibodies. Characteristic rhodamine**-**labeled cylindrical columns (red) in association with a Cld4^+^ chromogranin A^+^ cells (closed arrowheads) and a Cld4^−^ chromogranin A^−^ cells (arrows) are indicated. Bar, 20 µm. Asterisks (*) indicate luminal space. (B, C) Single cell suspensions of the small intestinal cells from control mice and those orally administered with fluorescein-dextran 30 minutes before were multi-color analyzed with FACS. The expression profiles of fluorescein-dextran in Cld4^–^ (dark blue boxes) and Cld4^+^ (red boxes) cells (B) and in Cld4^–^UEA-1^+^ (orange boxes), Cld4^+^UEA-1^+^ (yellow boxes), Cld4^–^UEA-1^–^ (light blue boxes) and Cld4^+^UEA-1^–^ (pink boxes) cells (C) of a PI^–^ CD45^–^ Ter119^–^ EpCAM^+^ cell gate are indicated. Black and green lines in the histograms indicate the profiles of control and dextran-administered mice, respectively. Percentages of fluorescence-positive populations are shown. The results are representative of at least three independent experiments.

Exocrine goblet cells are reported to be capable of capturing luminal antigens and delivering them to lamina propria [42], and therefore we investigated the antigen-capturing capacity of enteroendocrine cells with the use of rhodamine-conjugated dextran (10 kDa) as a model antigen. In 30 minutes following oral administration, the luminal surface of the intestinal epithelial cells was coated with dextran without diffuse invasion into the epithelial cellular sheets; however, characteristic cylindrical dextran columns projecting through the villus epithelium into the lamina propria were observed sporadically (Figure 6A). Some of the dextran columns coincided with Cld4^−^CgA^−^ epithelial cells, most likely representing goblet cells (Figure 6A). However, we also observed that a portion of the dextran columns coincided with spindle-shape CgA^+^Cld4^+^ epithelial cells (Figure 6A), although not all Cld4^+^ cells were associated with dextran columns at a given time (30 minutes) after dextran administration. FACS analysis confirmed that about 23% of Cld4^–^ and 32% of Cld4^+^ epithelial cells were labeled with fluorescein after fluorescein–dextran administration, whereas no staining was detected in the epithelial cells from untreated mice (Figure 6B). In the Cld4^–^ cell population, the strong fluorescein staining was confined to UEA1^+^ cells representing goblet cells and M cells, with only marginal staining in UEA1^−^ absorptive epithelial cells (Figure 6C). Within the Cld4^+^ enteroendocrine cell population, the vast majority (88.5%) of UEA-1^+^ cells revealed a strong fluorescein staining, although only a minor proportion (17.4%) of UEA-1^–^ cells representing K cells showed a marginal staining, if any (Figure 6C). These results collectively suggest that a proportion of enteroendocrine cells are capable of efficiently capturing luminal antigens, with the exception of K cells.

## Discussion

Enteroendocrine cells comprise a very small population of gut epithelial cells that are scattered solitarily throughout the gastrointestinal tract, and the physical isolation of these cells has been hampered by the lack of specific cell surface markers. In the current study, we found that CgA^+^ enteroendocrine cells in small intestine rather specifically express a Cld family member, Cld4, diffusely and strongly on the cell surface as revealed by immunostaining. Flow cytometric analysis using the rat anti-Cld4 monoclonal antibody (HKH-189) that recognizes the extracellular portion indicated that approximately 8% of total small intestinal epithelial cells expressed Cld4, and that the isolated Cld4^+^ cells exclusively exhibited the expression of the genes associated with enteroendocrine cells, such as *Chga*, *Ffar1*, *Gpr119*, and *Ffar4*. The proportion could be an overestimate for enteroendocrine cells, because the Cld4^+^ cell population may also include other minor epithelial cells such as FAE in Peyer’s patches and villous tip enterocytes [31]–[33]. We confirmed that a portion of these cells expressed Cld4 locally at TJs as well as laterally. However, we presume that such epithelial cells expressing a relatively low level of Cld4 may be hardly detected with FACS analysis because low-residual *Cldn4* transcripts could be detected in the Cld4^−^ cell population. Thus, in practice, it is strongly suggested that Cld4 serves as a specific cell surface marker covering entire enteroendocrine cells, providing a useful means for the isolation of this minor epithelial cell population as viable cells.

It has been reported that UEA-1 binds to a portion of enteroendocrine cells [15] and, in agreement, FACS analysis revealed that the Cld4^+^ epithelial cells consisted of UEA-1^+^ and UEA-1^−^ populations, both of which comparably expressed *Chga*. The Cld4^+^UEA-1^−^ cell population almost exclusively expressed *Gip*, with undetectable level of *Cck* or *Sct*, thus representing K cells. The population expressed lower levels of *Gcg* and *Pyy*, and it remains to be determined whether this was attributable to a minor contamination of UEA-1^low^ cells, or to overlapping hormone expression in a single cell type as suggested previously [17], [40], [41]. On the other hand, the Cld4^+^UEA-1^+^ cell population contained abundant transcripts of a series of hormones, including *Gcg*, *Pyy*, *Cck*, *Sct*, and *Tph1*, but no detectable *Gip*. Thus, although the cell population was much smaller than the Cld4^+^UEA-1^−^ cell population, it was suggested that the Cld4^+^UEA-1^+^ cell population comprises functionally heterogeneous enteroendocrine cells, including L cells. The results of gene expression analysis were confirmed at the protein level with immunostaining analysis; GIP expression was associated with Cld4^+^UEA-1^−^ cells, whereas GLP-1 expression was detected with Cld4^+^UEA-1^+^ cells with a minor overlap expression. The combination of rat anti-Cld4 monoclonal antibody (HKH-189) and UEA-1 thus provides an effective means to isolate K cells and other enteroendocrine cell populations, including L cells, separately from the intestinal epithelium. Introduction of additional markers may enable further elaboration of the individual enteroendocrine cell types.

Although M cells present in the FAE of Peyer’s patches are specialized for capturing luminal antigens and delivering them to lamina propria to initiate mucosal immunity [43], a recent report indicated that exocrine goblet cells are also capable of doing so [42]. Our current study indicates that Cld4^+^CgA^+^ enteroendocrine cells have an activity of taking up luminal materials as well. The antigen-capturing activity was found mainly in the minor Cld4^+^UEA-1^+^ cells population, with little activity in the Cld4^+^UEA-1^−^ K cells. Although enteroendocrine cells apparently form ZO-1^+^Cld4^+^ TJs with adjacent absorptive epithelial cells, it is unclear whether the luminal antigen is delivered through a transcellular route like M cells [44], or via a paracellular route due to the permissive TJs as implicated in goblet cells [42], [45]. Because our current results indicate that the fluorescein-labeled dextran could be detected in both goblet and enteroendocrine cells by FACS analysis, we presume that luminal antigens may be transferred into basolateral sites through a transcellular route of these cells. It also remains to be investigated whether such an activity of enteroendocrine cells is linked to the initiation of antigen-specific mucosal immunity, or related to any other biological functions. Nonetheless, Cld4 may be a potential target for the delivery of mucosal vaccines or peptide drugs [34]–[36]. In conclusion, our current results indicate that Cld4 is a new marker of enteroendocrine cells and provides a useful means for the study of their development and function.

## Materials and Methods

### Ethics

This study was conducted according to the principles expressed in the Declaration of Helsinki. The study was approved by Animal Research Committee, Graduate School of Medicine, Kyoto University (MedKyo13045).

### Mice

8-week-old to 10-week-old C57BL/6 WT female mice were purchased from Japan SLC and kept for at least 1 week before experiments in our animal room. *Cldn4* ^−/−^ mice were generated as described [29]. Mice were maintained in specific pathogen**-**free conditions at the Kyoto University Laboratory Animal Center in accordance with university guidelines. Mice were euthanized by cervical dislocation and used for experiments.

### Histological Analysis

Histological analysis was performed as described, with some modifications [46], [47]. For intestinal villi, tissue samples were fixed with 10 % formalin (Wako) for 16 hours at 4°C, followed by dehydration with 10% and 20% (wt/vol) sucrose solution before being frozen with liquid nitrogen in Tissue**-**Tek OCT compound (Sakura). Unless otherwise noted, cryostat sections (6 µm) were blocked with 2% (vol/vol) fetal calf serum in phosphate-buffered saline (PBS) for 30 minutes at room temperature, followed by being incubated with primary antibodies or reagents for 16 hours at 4°C. Samples were then washed with 0.1% PBS-Tween and PBS, each for 5 minutes, followed by staining with secondary reagents for 30 minutes at room temperature. Samples were washed with PBS twice and then counterstained with DAPI (Sigma**-**Aldrich) for 10 minutes at room temperature to visualize the nucleus. Finally, samples were washed with PBS twice, mounted in Mowiol (Calbiochem), and examined under a fluorescence photo microscope (Axiovert 200M; Carl Zeiss).

### Flow Cytometry Analysis and Cell Sorting

FACS analysis and cell sorting were performed as described previously [48]. In brief, the small intestine was cut longitudinally and washed in cold PBS. Then, a 2-cm piece of intestine was cut and incubated in 2% (vol/vol) fetal calf serum in RPMI 1640 (Wako) containing 0.5 mM EDTA (Nacalai Tesque) for 15 minutes at 37°C. Cells were filtered with a 70-μm cell strainer (Becton Dickinson) and blocked with Fc Block (2.4G2; Becton Dickinson) for 5 minutes at room temperature, followed by staining antibodies and reagents for 30 minutes at 4°C. Dead cells were excluded by propidium iodide (PI) staining. Epithelial cell fraction was defined by PI^−^CD45^−^TER119^−^EpCAM^+^ gating and biotinylated HKH-189 was used for Cld4 staining. Samples were analyzed by FACSCanto (Becton Dickinson) or sorted by FACSAria (Becton Dickinson).

### Antibodies

The antibodies and reagents used for immunohistological and flow cytometry analyses were as follows: purified or biotinylated rat anti**-**Cld4 mAb (HKH**-**189) [28]; fluorescein isothiocyanate**-**conjugated or biotinylated UEA**-**1 (Vector Laboratories); phycoerythrin (PE)**-**conjugated rat anti**-**CD45 mAb (30**-**F11) (Becton Dickinson); PE**-**conjugated rat anti**-**TER119 mAb (TER-119) (eBioscience); PE-Cy7–conjugated rat anti**-**EpCAM mAb (G8.8) (Biolegend); purified rabbit anti**-**chromogranin A pAb (ImmunoStar); purified rabbit anti**-**GIP pAb (Abbiotec); purified rabbit anti**-**GLP**-**1 mAb (EPR4043) (Epitomics); purified rabbit anti**-**ZO**-**1 pAb (Invitrogen); purified rat anti**-**ZO**-**1 mAb (R26.4C) (Developmental Studies Hybridoma Bank); purified rabbit anti**-**Cld3 pAb (Abcam); purified rabbit anti-Cld4 pAb (invitrogen); purified rabbit anti-Cld10 pAb (invitrogen);Cy3**-**conjugated anti**-**rat IgG (Jackson ImmunoResearch); Alexa Fluor 488–conjugated or Alexa Fluor 647**–**conjugated anti**-**rabbit IgG (Invitrogen); and allophycocyanin**-**conjugated streptavidin (Becton Dickinson).

### Quantitative Reverse-Transcription Polymerase Chain Reaction

RNA was extracted from isolated cells with Trizol (Invitrogen), followed by synthesis of cDNA with SuperScript III (Invitrogen). Gene expression level was assayed by real**-**time polymerase chain reaction (PCR) using QuantiTect STBR Green PCR mix (Qiagen) on a LyghtCycler 480 Real**-**Time PCR System (Roche). The transcripts of each gene were normalized to those of cyclophilin. The following primer sequences were used: *CgA*, (sense) 5′**-**CAGGCTACAAAGCGATCCAG**-**3′ and (antisense) 5′**-**GCCTCTGTCTTTCCATCTCC**-**3′; *Gip*, (sense) 5′**-**CAGGTAGGAGGAGAAGACCTCAT**-**3′ and (antisense) 5′**-**CCTAGATTGTGTCCCCTAGCC**-**3′; *Gcg*, (sense) 5′**-**CACGCCCTTCAAGACACAG**-**3′ and (antisense) 5′**-**GTCCTCATGCGCTTCTGTC**-**3′; *Ffar1*, (sense) 5′**-**TAGGACTGGCTTCTGGTGCT**-**3′ and (antisense) 5′**-**CTGCAGAGAAAAGAATGTCACAA**-**3′; *Gpr119*, (sense) 5′**-**CCAGGAATCGTGGTCCAG**-**3′ and (antisense) 5′**-**TGCATGTTCTTGAGAGAAGTCC**-**3′; *Ffar4*, (sense) 5′**-**TTCTGGGGCTCATCTTTGTC**-**3′ and (antisense) 5′**-**GCCACCAGCACTAGAGCAC**-**3′; *Cck*, (sense) 5′**-**TGATTTCCCCATCCAAAGC**-**3′ and (antisense) 5′**-**GCTTCTGCAGGGACTACCG**-**3′; *Sct*, (sense) 5′**-**GCTGTGGTCGAACACTCAGA**-**3′ and (antisense) 5′**-**GAGACAGGGACCCATCCAG**-**3′; *Pyy*, (sense) 5′**-**CCTACCCTGCCAAACCAG**-**3′ and (antisense) 5′**-**GGACATCTCTTTTTCCATACCG**-**3′; *Tph1*, (sense) 5′**-**CACAGTTCAGATCCCCTCTACA**-**3′ and (antisense) 5′**-**GAACGTGGCCTAGGAGTTCA**-**3′; *Cldn3*, (sense) 5′**-**TGGGAGCTGGGTTGTACG**-**3′ and (antisense) 5′**-**CAGGAGCAACACAGCAAGG**-**3′; *Cldn4*, (sense) 5′**-**GGCGTCTATGGGACTACAGG**-**3′ and (antisense) 5′**-**GAGCGCACAACTCAGGATG**-**3′; *Tjp1*, (sense) 5′**-**CGCGGAGAGAGAGACAAGATGT**-**3′ and (antisense) 5′**-**TCCTCCATTGCTGTGCTCTT**-**3′; and *cyclophilin*, (sense) 5′**-**GACGAAGGTAGCCAGTCACAAG**-**3′ and (antisense) 5′**-**AATCAGGCCTGTGGAATGTGAG**-**3′.

### Oral Challenge of Dextran

C57BL/6 WT mice were administered oral 5 mg/500 µl of 10-kDa tetramethylrhodamine or fluorescein**-**conjugated lysine-fixable dextran (Life Technologies). Thirty minutes after oral challenge, the small intestine was examined by immunohistochemistry or FACS as described above..

### Statistical Analysis

Data are presented as mean values ± standard deviation. Statistical significance was determined with the Student *t* test or ANOVA.

## Supporting Information

Figure S1 **Cld4 signals detected by independent anti-Cld4 antibodies were absent in** ***Cldn4^−/−^*** **mice.** Small intestine of WT and *Cldn4^−/−^* mice were immunostained with anti-Cld4 polyclonal antibody or monoclonal antibody (HKH-189) (green) and DAPI (white). Bars, 50 µm.
(TIF)
