# Supplementary figures and images for: Enteroendocrine Cells Are Specifically Marked by Cell Surface Expression of Claudin-4 in Mouse Small Intestine
Source: PLoS One. 2014 Mar 6;9(3):e90638. doi: 10.1371/journal.pone.0090638 (PMC3948345; doi:10.1371/journal.pone.0090638)

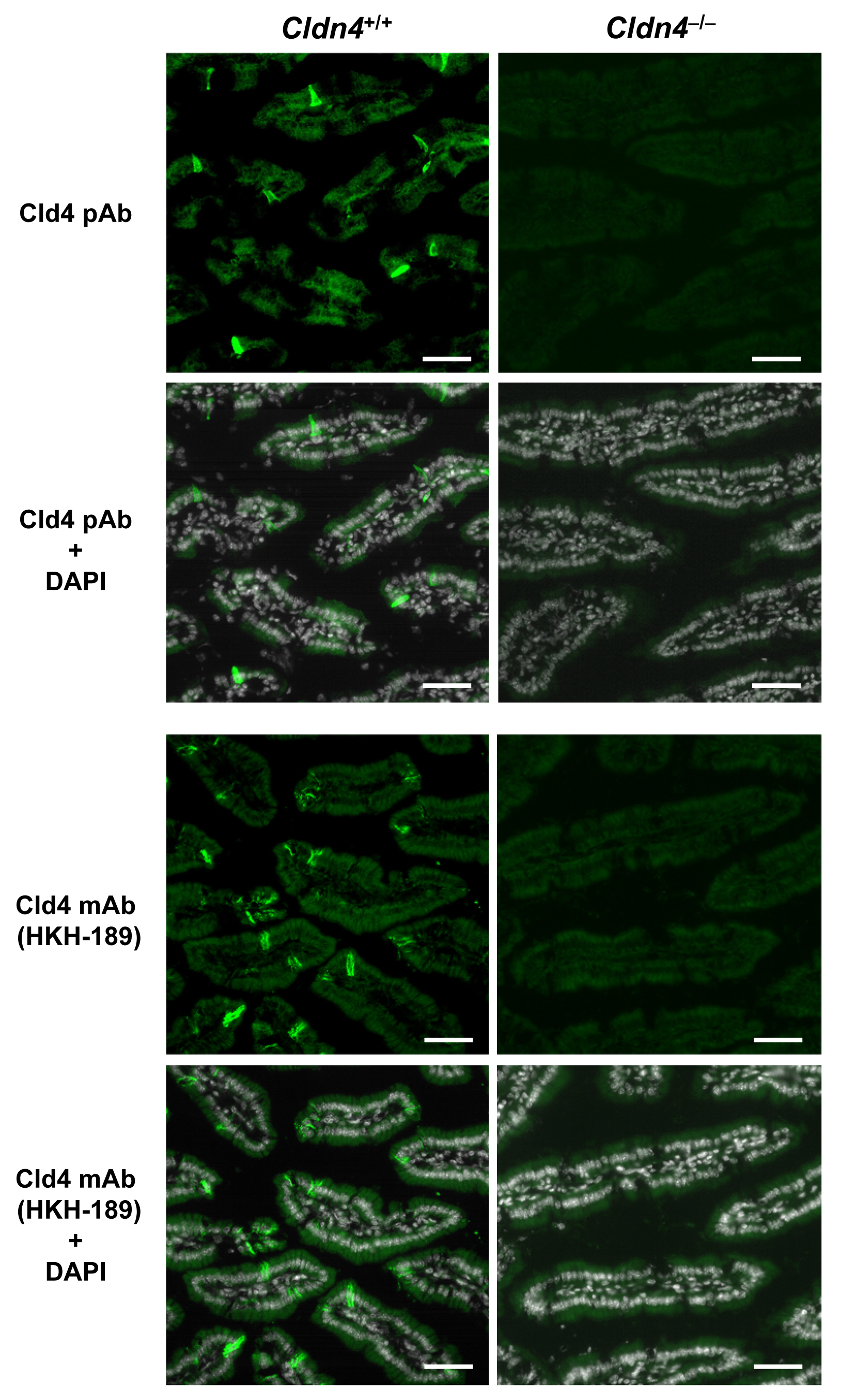

Supplement: Figure S1 — Cld4 signals detected by independent anti-Cld4 antibodies were absent in Cldn4−/− mice. Small intestine of WT and Cldn4−/− mice were immunostained with anti-Cld4 polyclonal antibody or monoclonal antibody (HKH-189) (green) and DAPI (white). Bars, 50 µm. (TIF) [file pone.0090638.s001.tif]
